# Trends of Phase I Clinical Trials of New Drugs in Mainland China Over the Past 10 Years (2011–2020)

**DOI:** 10.3389/fmed.2021.777698

**Published:** 2021-12-17

**Authors:** Chen Chen, Ning Lou, Xin Zheng, Shasha Wang, Haizhu Chen, Xiaohong Han

**Affiliations:** ^1^Clinical Pharmacology Research Center, Peking Union Medical College Hospital, State Key Laboratory of Complex Severe and Rare Diseases, NMPA Key Laboratory for Clinical Research and Evaluation of Drug, Beijing Key Laboratory of Clinical PK & PD Investigation for Innovative Drugs, Chinese Academy of Medical Sciences & Peking Union Medical College, Beijing, China; ^2^Department of Clinical Laboratory, National Cancer Center/National Clinical Research Center for Cancer/Cancer Hospital, Chinese Academy of Medical Sciences & Peking Union Medical College, Beijing Key Laboratory of Clinical Study on Anticancer Molecular Targeted Drugs, Beijing, China; ^3^Department of Medical Oncology, National Cancer Center/National Clinical Research Center for Cancer/Cancer Hospital, Chinese Academy of Medical Sciences & Peking Union Medical College, Beijing Key Laboratory of Clinical Study on Anticancer Molecular Targeted Drugs, Beijing, China

**Keywords:** phase I clinical trial, innovative drugs, phase transition, therapeutic area, geographical distribution

## Abstract

**Background:** In recent years, the number of clinical trials initiated in China has increased rapidly. The aim of this study was to overview the changing landscape of phase I clinical trials in mainland China from 2011 to 2020.

**Methods:** We analyzed phase I clinical trials registered on 3 websites including the Chinese Clinical Trial Registry, ClinicalTrials.gov, and the China National Medical Products Administration Center for Drug Evaluation platform.

**Findings:** A total of 2,842 phase I clinical trials were posted from January 1, 2011, to December 31, 2020. The overall number of clinical trials for innovative drugs was 1,497, accounting for half of all the phase I clinical trials (53%). Among these 1,486 innovative drug clinical trials, 924 were newly tested drugs with an average annual growth rate of 59%. Biological drug research increased significantly from 22.6% during 2011–2015 to 33.3% during 2016–2020. These principal investigators (PIs) of these clinical trials were mainly from Beijing (*n* = 871), followed by Shanghai (*n* = 496) and Jiangsu (*n* = 281). As for the therapeutic area of phase I clinical trials, cancer took up the most percentage of all the clinical trials (35%), followed by infectious disease (9%), nervous system disease (9%), etc. Most phase I clinical trials are conducted on healthy volunteers (*n* = 1,642, 57.8%), some cancer drugs are conducted in patients with cancer (*n* = 846, 29.8%), and only a few clinical trials were conducted in the elderly (*n* = 7). Among these clinical trials of the newly tested innovative drugs, the first in human (FIH) clinical trials accounted for 82% (744), and the First in Chinese (FIC) clinical trials only took up 18% (167). Only a small number of drugs could be made the transition to phase II (*n* = 207, 22%). In addition, despite the number of newly tested drugs during 2011–2015 (*n* = 163) was much less than that in 2016–2020 (*n* = 761), the percentage of drugs that could enter into phase II clinical trials in 2011–2015 (34%) was higher than that in 2016–2020 (20%).

**Conclusion:** In the past 10 years, the development of phase I clinical trials has achieved great progress in mainland China due to the novel design and drug innovation policy. Nevertheless, future efforts are needed to make for improving the phase transition success rate of innovative drugs.

## Introduction

In the past 10 years (2011–2020), following the opportunities created by the availability of many new drugs and substantial increase in the number of patients available to be enrolled in trials, the number of registered clinical trials is increasing (1).

Three authoritative clinical trials registration databases are an important resource to view and access clinical trials registration data including the Chinese Clinical Trial Registry (ChiCTR) that was established in 2005 and was assigned to be the representative of China to join the WHO International Clinical Trials Registry Platform (ICTRP) in 2007 (2), the ClinicalTrials.gov that was launched in 2000 and was managed by the National Library of Medicine (3), and the China National Medical Products Administration (NMPA), the former name is the China Food and Drug Administration (CFDA) Registration and Information Disclosure Platform for Drug Clinical Studies, which was established in 2013 (4). Analyzing clinical trials registration data can illuminate important trends over time.

A phase I trial represents the critical transition of a novel compound from the preclinical to clinical stage, and thus provides the foundation for an efficacious drug development program (5). Nowadays, phase I clinical trials have evolved from the traditional role of dose and toxicity-finding studies to innovative study designs that match patients with study agents and rely on the model-informed drug development (MIDD), thus increasing the potential of clinical efficacy, even in the early dose-escalation setting (6). Given that maximum tolerated dose in phase I clinical trials was different between the people of Japan and the West in some cytotoxic anticancer drugs due to the intrinsic and extrinsic ethnic factors (7), some differences in phase I clinical trials may also exist between China and other countries. Therefore, analyzing phase I clinical trials registration data of China can not only give us information about the development of novel therapeutic drugs in China over the past decades but also illuminate the trends and developmental directions of medicine in the future years. However, the previous time-trend analysis of clinical trials research and development focused on all the phases of a specific research field such as oncology.

In this study, we evaluated the trends over the past decades of phase I clinical trials in China from 2011 to 2020 and aimed at providing insight into the future direction of novel therapeutic drugs, identifying the unmet clinical needs, and providing essential supportive data for investigators, pharmaceutical enterprises and policymakers.

## Methods

### Data Sources

Clinical trials registered in the Chinese Clinical Trial Registry (ChiCTR) (http://www.chictr.org.cn) and ClinicalTrials.gov (https://clinicaltrials.gov/) websites with the first posted date from January 1, 2011, to December 31, 2013, were collected for analysis since the data on the China National Medical Products Administration (NMPA) Center for Drug Evaluation (CDE) website (http://www.cde.org.cn/) are only available since 2013. Due to Registration on the CDE platform being mandatory for new drug registration trials in China according to the NMPA from its establishment on September 6, 2013, the data from the CDE registration platform were all collected.

### Study Design

As depicted by the FDA, phase I studies are designed mainly to investigate the safety/tolerability (if possible, identify MTD), pharmacokinetics, and pharmacodynamics of an investigational drug in humans (8). We searched ClinicalTrials.gov by selecting “phase I” in the term of “phase”, filling the “first-posted date” blank of from January 1, 2011, to December 31, 2013, and restricted the country to China. Using this search strategy, 246 trials were identified from the website of ClinicalTrials.gov. A total of 196 phase I clinical trials (interventional studies) with registration dates between January 1, 2011, and December 31, 2013, that were recorded in ChiCTR were selected. Meanwhile, the phase I clinical trials (*n* = 3,217) were screened from all clinical trials registered on the CDE website were collected for further selection.

Then, the duplicated clinical trials were preliminarily identified based on reporting the same sponsor, drug, and study name. In addition, other clinical trials do not intend for pharmacokinetics study including these for new therapeutic apparatus, new surgical approach, bioequivalence studies, clinical trials initiated by researchers or company that was registered as phase I after the drug is on the market that was also excluded from this study.

**Figure 1:**
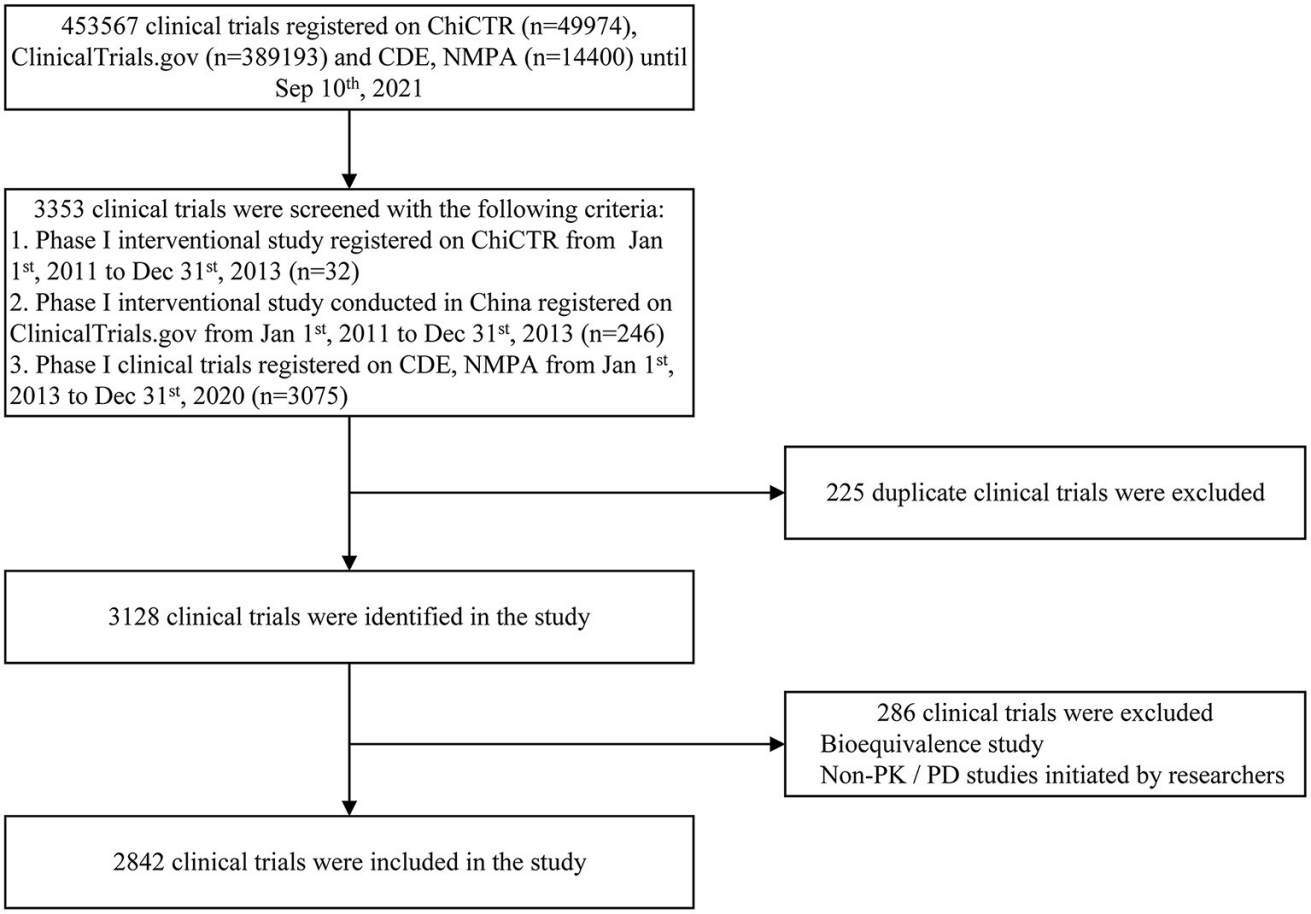
Flow diagram of the phase I clinical trial selection process.

Three investigators (NL, CC, and SSW) independently extracted data study-by-study and excluded trials that did not meet the aforementioned criteria. Then two other investigators (NL and SSW) identified duplicated trials. When consensus was not reached by discussion, all investigators were consulted for the final decision. Finally, 2,842 clinical trials were included in the final analysis (Figure 1).

### Statistical Analysis

We used R software, version 3.6.2 (http://www.r-project.org/) for data process and analysis. For descriptive analyses, the number was used for qualitative variables. We analyzed the 10-year trends in our selected indicators, including the number of phase I trials, the number of phase I trials involving different drug types, the number of leading clinical trial units, the number of trials entering into phase II trials, the number of innovative drugs, and the number of newly tested trials. The annual rate of change was calculated for each indicator. The first posting year of a study was defined by the registered date.

## Results

### Time Trends of Phase I Clinical Trials

#### Annual Change of Phase I Clinical Trials

**Figure 2:**
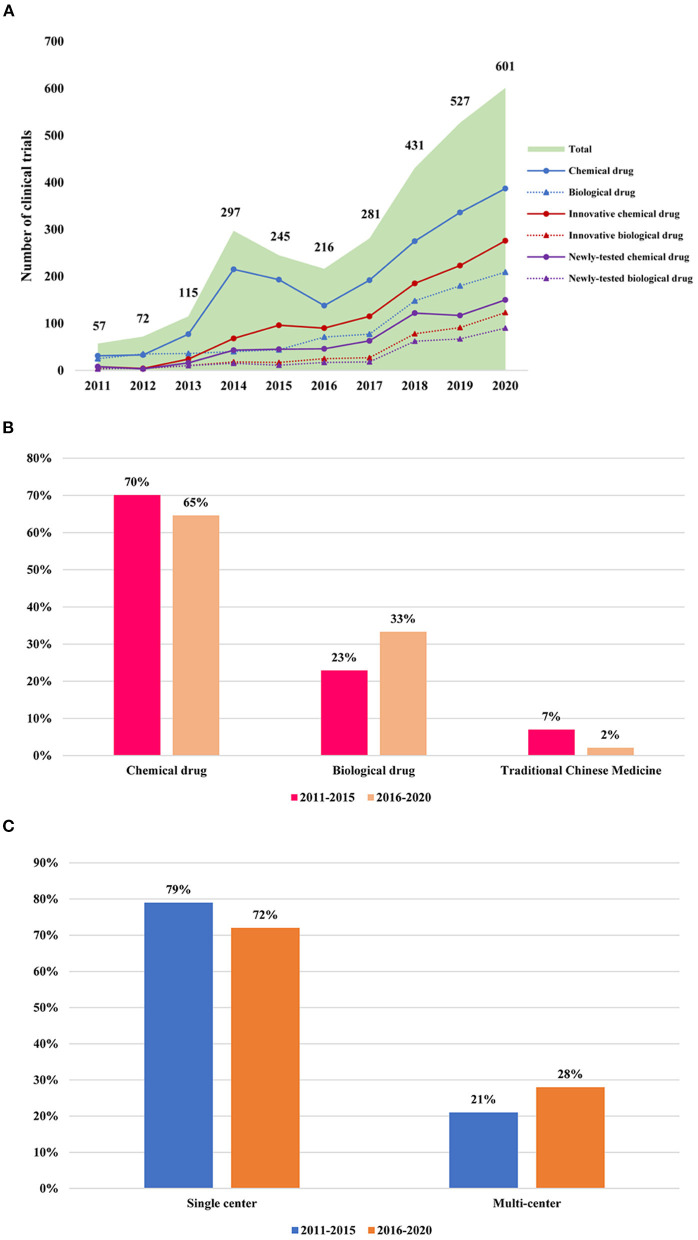
Change of phase I clinical trials in mainland China during 2011–2020. **(A)** Annual change of phase I clinical trials in mainland China during 2011–2020. The data cutoff date was December 30, 2020; **(B)** The changes of the chemical drug, biological drug, and traditional Chinese medicine every 5 years; **(C)** The changes of a single center and multicenter design every 5 years.

A total of 2,842 phase I clinical trials registered on the three public websites from January 1, 2011, to December 31, 2013, in the ClinicalTrials and ChiCTR and from 2013 in CDE were available for analysis in this study. There were 57 in 2011, 72 in 2012, 115 in 2013, 297 in 2014, 245 in 2015, 216 in 2016, 281 in 2017, 431 in 2018, 527 in 2019, and 601 in 2020 (Figure 2A). The annual number of initiated phase I clinical trials increased over time, with an average annual growth rate of about 37%. A notable increase in the number of clinical trials was seen in 2018, with an increase of 53.4% over the number initiated in 2017 (Figure 2A).

Innovative drugs refer to drugs with a clinical value containing new compounds with a clear structure and pharmacological effects (9, 10). Combined with the classification of chemical drugs and biological products in the “Administrative Measures for Drug Registration,” the innovative drugs discussed in this article mainly refer to category 1 chemical drug (also named as “chemical innovation drugs”), the original category of 1.1 and 1.2 chemical drugs, and category 1 biological drugs (also named as “biological innovation drugs”). Considering that traditional Chinese medicines have certain differences from chemical drugs and biological products, they were classified as noninnovative drugs in this study.

The number of clinical trials for innovative drugs was 1,486, accounting for half of all phase I clinical trials (52%) (Figure 2A). In addition, the phase I clinical involving innovative drugs showed a slight increase from 2011 to 2017, whereas it increased rapidly in 2018, with a growth rate of 62% for chemical drugs and 186% for biological drugs (Figure 2A). Importantly, the proportion of clinical trials of innovative drugs is increasing annually (Figure 2A).

Among these 1,486 innovative drug clinical trials, only 911 were newly tested drugs. The number of newly tested drugs growth annually with an average growth rate of 59% and significant growth of newly tested drugs could be seen since 2017 with the growth rate of 66 and 34% for chemical drugs and biological drugs, respectively (Figure 2A).

#### Changes in the Type of Drug in Clinical Trials

It can also be seen that the proportion of clinical trials involving chemical drug research has decreased slightly from 70.4% in 2011–2015 to 64.5% during 2016–2020, while the proportion of clinical trials involving biological drug research increased significantly from 22.6% during 2011–2015 to 33.3% during 2016–2020. This phenomenon reflected the rapid development of biopharmaceuticals in recent years (Figure 2B).

#### Changes of Multicenter Design in Phase I Clinical Trials

Among these 2,842 registered in phase I clinical trials, the percentage of multicenter phase I clinical trial accounted for 28% of all phase I clinical trials in 2016–2020, which showed a slight increase of 7% compared with that during 2011–2016 (21%) (Figure 2C).

### Geographical Distribution of Phase I Clinical Trials

**Figure 3:**
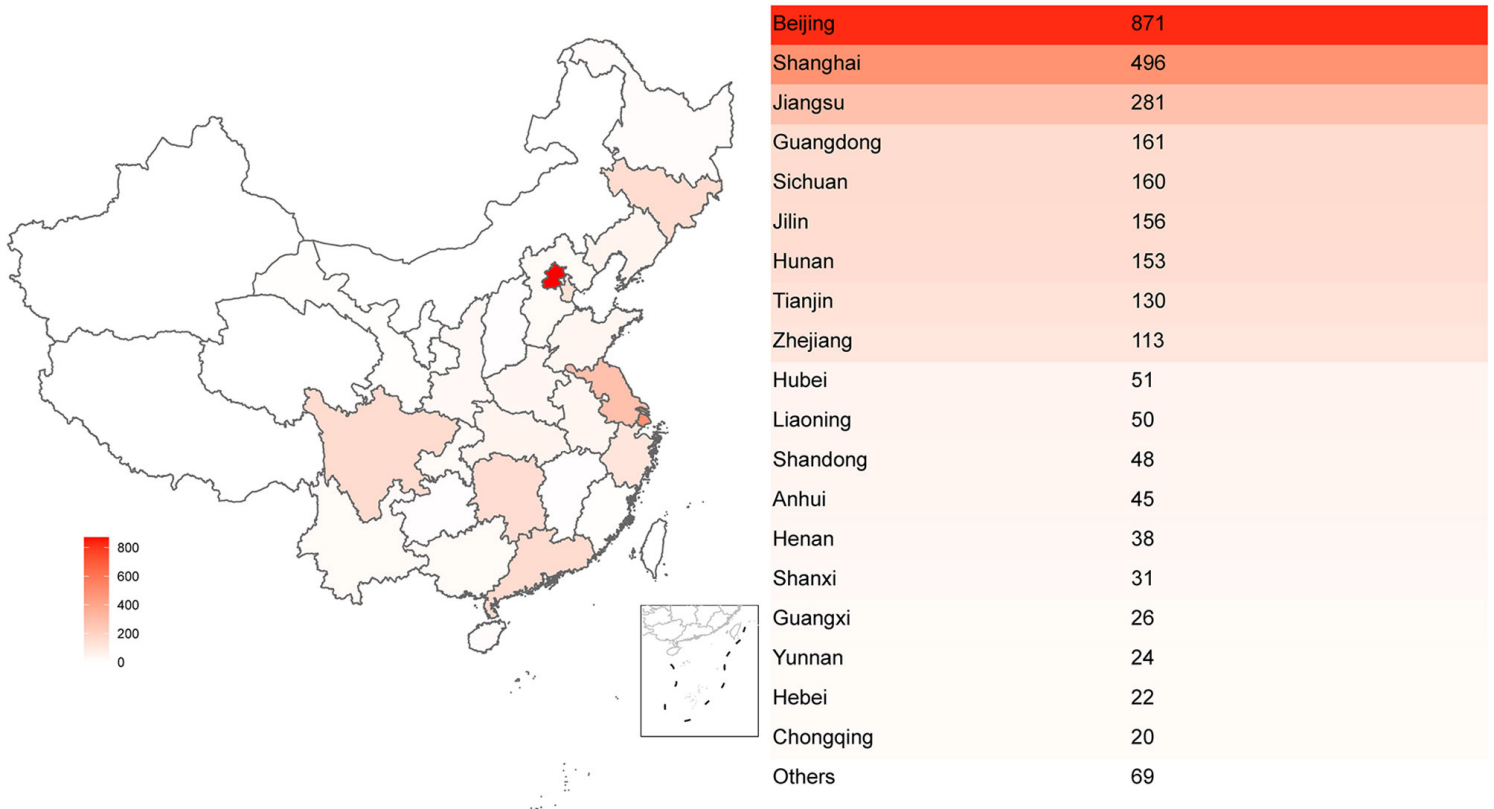
Geographical distribution of phase I clinical trials during 2011–2020.

Overall, the principal investigators (PIs) of these 2,842 phase I clinical trials were distributed at different provinces or municipalities across China. A total of 871 PIs of phase I clinical trials was initiated in Beijing, accounting for the most percentage (31%), followed by Shanghai (*n* = 496) and Jiangsu province (*n* = 281) with the percentage of 17 and 10%, respectively. On the contrary, the number of initiated phase I clinical trials are rare in northwest China (Gansu, Qinghai, Xinjiang) (Figure 3). This result showed a severe uneven geographical distribution across China.

### Therapeutic Areas of Phase I Clinical Trials

**Figure 4:**
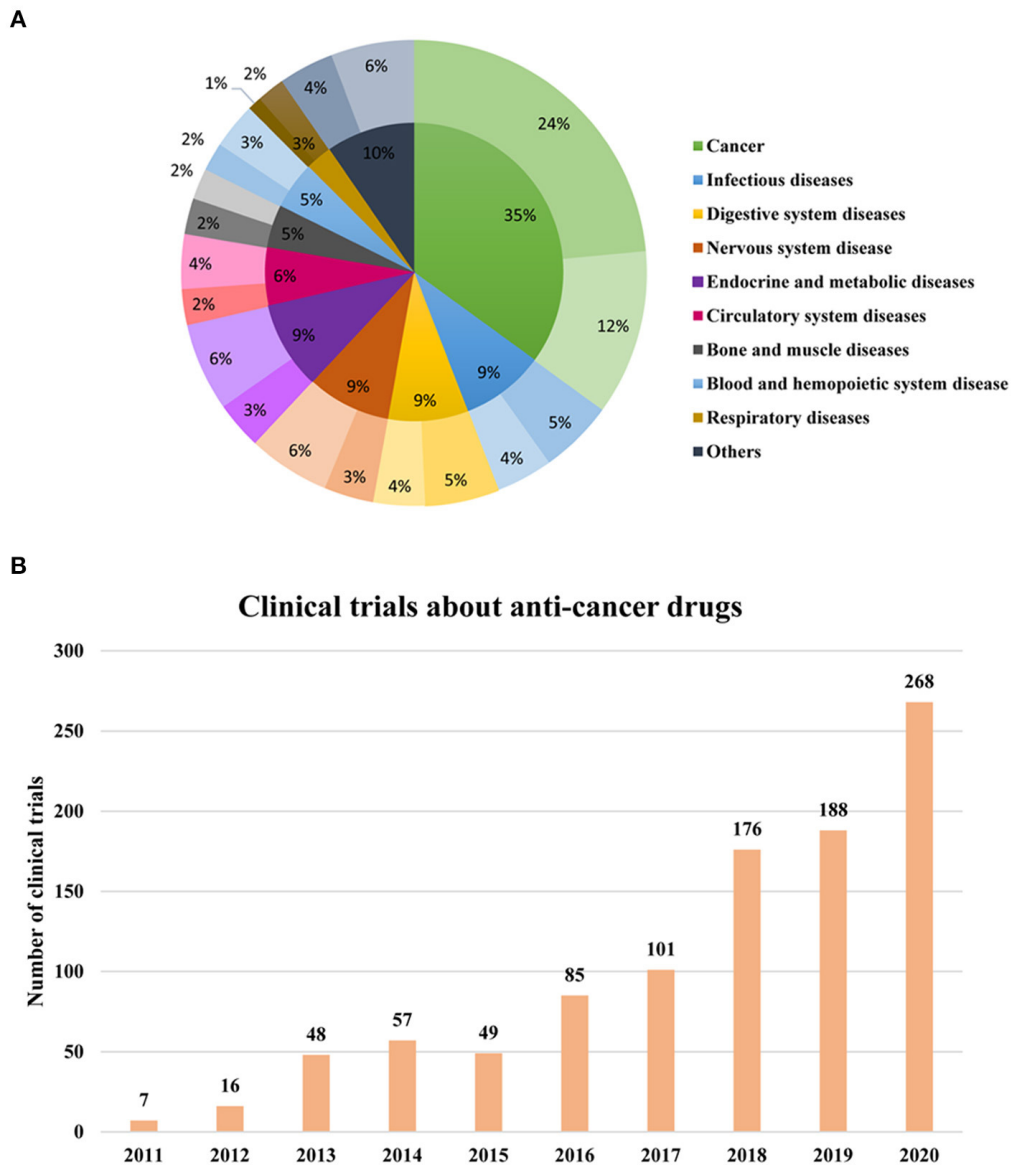
Therapeutic areas of phase I clinical trials during 2011–2020. **(A)** Therapeutic areas in phase I clinical trials during 2011–2020. The darkest of one color represented phase 1 clinical trials of both innovative and noninnovative drugs; The slighter color shows the phase 1 clinical trials of innovative drugs; The lightest color represented the clinical trials conducted on the noninnovative drug. **(B)** Annual change of phase I clinical trials about oncology during 2011–2020.

According to the disease classification standard of ICD-11, these 2,842 phase I clinical trials were classified into different treatment fields (Figure 4A). It mainly targets cancer (35%), infectious diseases (9%), nervous system disease (9%), digestive system diseases (9%), endocrine and metabolic diseases (9%), followed by circulatory system diseases (6%). Among the endocrine and metabolic diseases (243), diabetes (218) took up most of the clinical trials and the number of digestive diseases (*n* = 259) with hepatitis is 145, accounting for more than half of all the digestive diseases (56%). Among the 268 clinical trials of anti-infectious drugs, half of them were clinical trials of antibacterial drugs (135), which were followed by antivirus drugs (103), and only 30 clinical trials are about antifungal drugs. In 179 clinical trials of cardiovascular disease, the drug indications of the clinical trials are mostly related to hyperlipidemia (50), followed by hypertension (40).

Consistent with the overall phase I clinical trials therapeutic area, the top 3 therapeutic areas of phase I clinical trials for innovative drugs are cancer, digestive disease, and endocrine and metabolic disease. Moreover, it can be seen that clinical trials about anticancer drugs take up almost half of all the phase I clinical trials for innovative drugs (Figure 4A). In addition, it was observed that the clinical trials of anticancer drugs accounted for the largest percentage of all the phase I clinical trials every year compared with other indications and the proportion has reached 45% in 2020 (Figure 4B). The results indicated that the therapeutic areas of phase I clinical trials conducted in China are closely connected to the disease spectrum of Chinese residents in recent years.

### The Target Population of Phase I Clinical Trials

Given that the objectives and nature of the trial differ depending on the subject, the target population was described in this study (Table 1). Phase I trials were mostly conducted in healthy volunteers (*n* = 1,642, 57.8%), but some phase I trials, such as oncology, are conducted in the patients with cancer (*n* = 846, 29.8%), and 11.2% of phase I clinical trials were performed on patients with another kind of diseases (*n* = 318). Only seven clinical trials were conducted in the elderly and 29 studies recruited special populations such as patients with renal or liver function or obesity.

**Table 1:** The target population of phase I clinical trials.

**Target population**	**Healthy volunteers**	**Cancer patients**	**Other disease patients**	**The elderly**	**Special populations (Patients with renal or liver function, obesity)**
Number (%)	1642 (57.8%)	846 (29.8%)	318 (11.2%)	7 (0.2%)	29 (1.0%)

### Changes of Newly Tested Innovative Drugs

**Figure 5:**
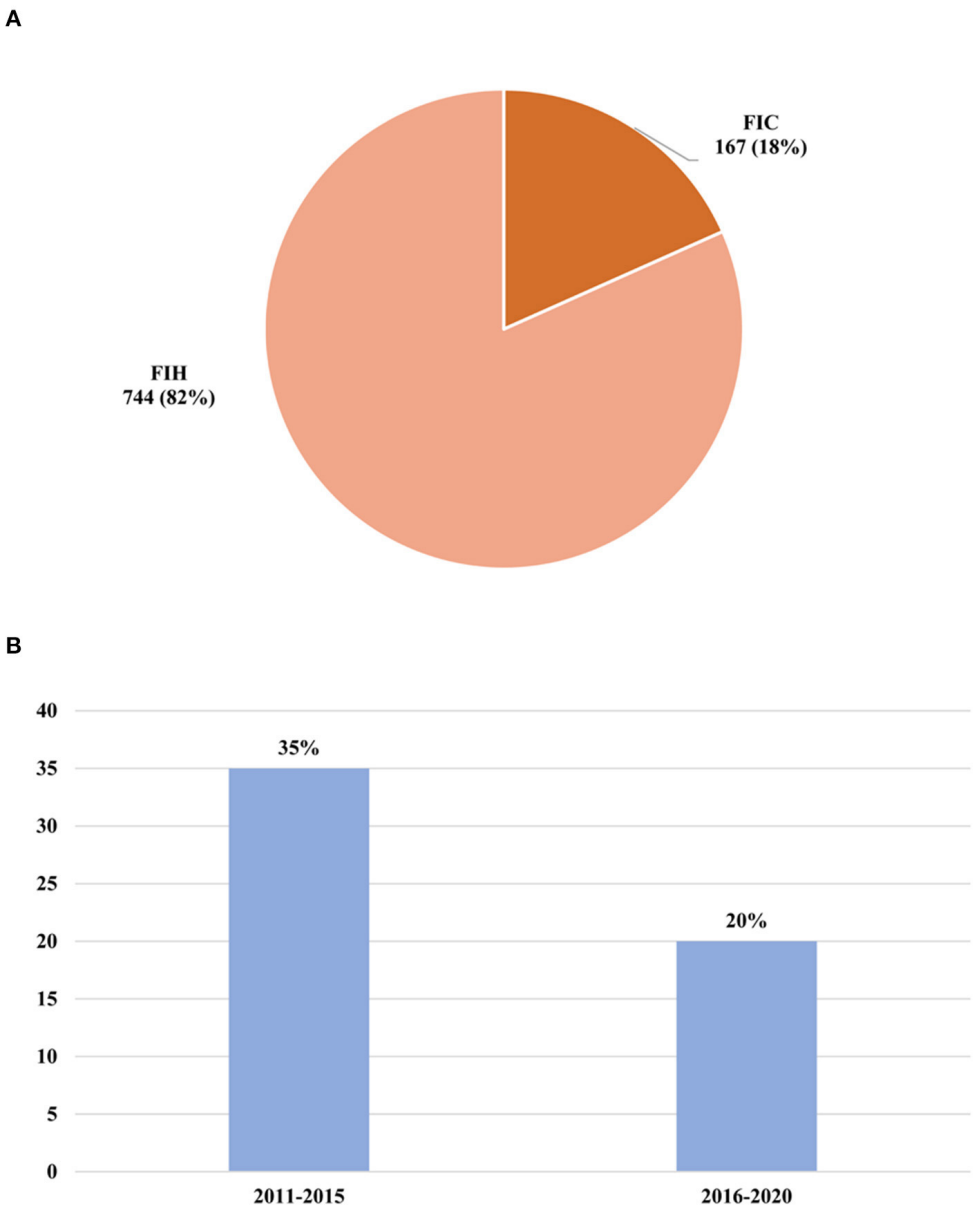
Changes of newly tested innovative drugs. **(A)** Percentage of FIH or FIC clinical trials of newly tested innovative drugs conducted in mainland China during 2011–2020. **(B)** Successful rate of phase transition of a newly tested innovative drug. FIH, first in human; FIC, first in China.

The percentage of “First in human” (FIH) phase I trials and “First in Chinese” (FIC) phase I trials of these innovative drugs were also investigated (Figure 5A). Among these clinical trials of newly tested innovative drugs, the FIH clinical trials accounted for 82% (744), and the FIC clinical trials only took up 18% (167).

Success rates for individual phases of the drug development process were determined by dividing the number that successfully advanced to the next phase by the total number advanced and suspended. In addition, we make the standard assumption that phase II/III was to be considered as phase II. It was found that only a small number of drugs could successfully be made the transition to phase II (*n* = 207). Of note, the number of newly tested drugs during 2011–2015 (*n* = 163) was much less than that in 2016–2020 (*n* = 761), while the percentage of drugs that could enter into phase II clinical trials in 2011–2015 (34%) was higher than that in 2016–2020 (20%) (Figure 5B). This result showed that despite the acceleration of drug research development domestic, the success rate of the drugs for approval for therapeutic use was further decreasing.

## Discussion

This study provided substantial information on the landscape of phase I clinical trials in mainland China. An increase in the number of initiated trials and newly tested innovative drugs in China illustrate the progress made from 2011 to 2020. The geographical distribution of leading clinical trial units, and changes in the therapeutic area, newly tested drugs, and phase transition success rate were analyzed to provide directions for people involved in the drug development process.

### Drug Research and Development Policy

From 2011 to 2020, the number of phase I trials in mainland China showed remarkable growth, with an average annual growth rate of 37%. These are also evidence that the Chinese new drug development system and clinical trial system have increasingly improved and developed. Since 2008, the Chinese Major New Drug Innovation Program for Chinese “Eleventh Five-Year Plan” (11), “Twelfth Five-Year Plan” (12), “Thirteenth Five-Year Plan,” (13) and other national-level policy plans have continuously supported the development of innovative drugs and the construction of the drug clinical trial platforms. These policies are aimed at addressing the serious lag in drug research and development in China and fostering more innovative drug companies and more internationally compatible clinical trial institutions with good clinical practice (GCP) regulatory systems and capabilities.

The NMPA has implemented a new priority examination and approval process since 2015, which emphasis approval based on clinical value. The China State Council released “Opinions on Reforming the examination and Approval System for Drugs and Medical Devices” on August 18, 2015, which was a milestone policy (14). Subsequently, a series of regulatory policies were enacted. In particular, the General Office of the Communist Party of China Central Committee and the General Office of the State Council issued “The opinions on deepening the reform of the examination and approval system to encourage the innovation of drugs and medical devices” on October 8, 2017 (15). In addition, since the restrictions on imported drug approvals were relaxed on October 10, 2017, the clinical trial data acquired abroad could be accepted (16). Moreover, the NMPA announced that clinical trials could start within 60 working days from the date of application if no negative and questionable review or opinion was received in 2018. This 60-day acquiescence system for new drug approval was further emphasized in the “Provisions for Drug Registration” issued in January 2020 (17). These improvements will encourage domestic and international enterprises to perform clinical trials of their drugs in China. Therefore, the number of clinical trials has increased significantly in China since 2017.

### Implications for Institutions/Investigators

Over the last 10 years, the phase I trials have evolved from single-site studies to increasingly multi-institutional efforts with the goal of expanding patient enrollment to make subjects more representative. However, in multi-institutional clinical trials, more than 3 participating sites enrolled patients, and slots in each cohort are allocated by the sponsor or filled on a competitive first-come-first-serve basis. Moreover, additional staff, resources, and frequent teleconferences among participating centers are needed to ensure real-time notification of adverse events and drug-related dose-limiting toxicities. These factors have led to greater reliance on clinical research organizations (CROs) to coordinate clinical trials management, to meet tight timelines, and to ensure uniformity of trial conduction across different centers. Furthermore, the need to accelerate patient recruitment led to the selection of clinical trial centers based on their ability to enroll rather than on their experience, which might result in more inexperienced investigators and clinical trial practices being involved in phase I clinical trials. Therefore, to ensure proper conduction of studies, compliance with the principles of GCP, and scientific rigor in data analysis and results reporting, all site investigators must participate regularly in teleconferences with the CRO to review relevant data of the patients and make the decision on dose-escalation strategies. As phase I clinical trials have more institutions involved, the establishment of an independent team of reviewers to objectively evaluate the performance of CROs and/or analyze trial data on an ongoing basis might be required.

### Development of Biopharmaceuticals

In recent years, the rapid development of biotechnology has promoted the development of biological drugs. A series of policies were released by the government to encourage the development of biomedicine. Since immune checkpoint therapy won the Nobel Prize in 2018 (18), immune checkpoint inhibitor monoclonal antibodies have gained much attention in China. However, the targets of drugs were limited to cytotoxic T lymphocyte-associated protein 4, programmed cell death 1, and its ligand in the Chinese biopharmaceutical firms. In contrast, the global immuno-oncology pipeline has broadened to novel targets, such as lymphocyte activation gene 3, V-domain Ig suppressor of T-cell activation, CD47, and the T-cell immunoglobulin and mucin domain-containing molecule 3 (19–21). It can be seen that the independent innovation ability of Chinese domestic biopharmaceutical companies needs to be improved.

Besides, despite cell therapy accounting for a small percent of all clinical trials, significant growth could be seen. Only three phase I clinical trials were registered in 2018 while the number increased to 12 in 2020. In February 2021, the Chinese National Health Commission replied to the relevant suggestion of the representatives of “Suggestions on the strategy of vigorously developing the health care industry to help healthy China” on the official website, clearly indicating support for stem cells, immune cells, and other research, transformation, and industrial development. Cell preparation products such as stem cells and immune cells have obvious drug properties (22). Once being approved, it can be widely used, which could not only ensure medical quality and safety but also be conducive to industrialization (22).

### Geographic Distribution

The data showed that the geographical distribution of the trial units in China is uneven. The regional economic level is the main factor that attributes to the uneven regional distribution of the number of trials. This geographical difference is evidence of the uneven distribution of clinical research medical resources in China. It is also likely to be attributed to the demand of government for the Chinese pharmaceuticals to fully play the leading role of major hospitals, with resource priority given to the exemplary role of top leaders. The Chinese Major New Drug Innovation Program issued the Notice on printing and distributing the requirements for the work of the construction of the major clinical drug evaluation technology platform for major new drugs on January 21, 2019 (23). This notice announced that major clinical trial centers played a leading role in new drug development, aiming at facilitating the formation of a highly motivating environment for medical innovation (23). Therefore, how to balance the equitable access to new drugs and the efficiency of pharmaceutical development remains to be an important topic worthy of exploration by policymakers.

### Trials Guided by Clinical Value in China

The disease spectrum in China has changed from acute infectious diseases to chronic noninfectious diseases. At present, stroke, ischemic heart disease, and chronic obstructive pulmonary disease are the top three killers of premature deaths among the Chinese (24, 25). It was reported that the three major causes of disability in China were musculoskeletal diseases, mental illnesses, and sensory organ diseases in 2017 (24). The incidence of infectious diseases and digestive system diseases is expected to decline slowly, while the incidence of chronic diseases such as diabetes will continue to explode (26–28). There are significant differences in drug structure between China and the globe. For example, the proportion of anti-infective drugs, digestive system drugs, and immunomodulators is too high (29, 30). The results partially reflected the difference between the Chinese current disease spectrum and the global spectrum. Compared with the global level, there is big room for growth in the field of diabetes, respiratory system, and degenerative diseases in China.

Phase I trials are mostly conducted in healthy volunteers, but some phase I trials, such as oncology, are conducted in patients with cancer. Depending upon the subject, the objectives and nature of the trial differ. In China, only a few studies were conducted in the elderly or special populations such as patients with renal or liver function. With the heavy growth of Chinese aging people, more attention was attached to the medication for the elderly. In the future years, more clinical trials will be conducted on the aged population.

### Development of Innovative Drugs

Great importance has been attached to pharmaceutical innovation in China. Planning guidelines such as “National Innovation-Driven Development Strategy Outline,” “Pharmaceutical Industry Development Planning Guide” and other planning guidelines all put forward goals for the development of innovative drugs and set up special projects (31, 32). Therefore, under the support of these national policies, the innovative drug has developed by leaps and bounds. The average annual growth rate of new drugs in the clinical trials has been 36%.

Trails of First in human are studies where an investigational medical product, previously assessed through *in vitro* or animal testing, is tested on human subjects for the first time (33). FIC means the investigational medical product had been conducted in other countries but was tested in China for the first time. From the perspective of global contribution, the FIH and FIC phase I clinical trials were investigated in this study. For the last 10 years, the FIH phase I clinical trials of the innovative drug took up the most percentage. This means that most Chinese domestic pharmaceutic companies choose to conduct phase I clinical trials in China, while some choose Australia. Some big international pharmaceutic companies also choose China as a target country to conduct phase I clinical trials. These results suggested that clinical trial management is becoming more and more standardized and international, and China has the capability of undertaking phase I first in human research.

In order to know how many drugs have the potential to be approved, the phase transition success rate was analyzed. However, in contrast to the annual growth trend, the phase I transition success rate during 2016–2020 (20%) was lower than that in 2011–2015 (34%), the lower phase I success rates during 2016–2020 may also be attributed to the changes of phase I clinical trials design and objectives. In recent years, efficacy has been added to the study of phase I (5). The designs of phase I clinical trials can maximize their potential to yield preliminary evidence of activity by incorporating appropriate efficacy endpoints, and the application of MIDD to predict the result of phase II clinical trials could also avoid conducting trials that are likely to fail, which might result in the decreased phase transition. In addition, the delayed reporting of phase II clinical trials may be another reason for the decrease of phase transition rate.

According to a previous report, success rates by disease area for phase I ranged from 40.9 to 71.6%, with the average for all the disease indications coming in at 52.0% according to the Biomedtracker database (34). In addition, this ratio is approximately 70% in US FDA (35). So as can be seen, the average phase transition success rate in China (22%) was much lower than that in America. From the results of this study, innovative drugs are in a rapid development stage in China, and a large number of phase I studies have been carried out. Among them, tumor drugs account for the majority. Since phase I of anticancer drugs usually take a long time from start to finish than other drugs, many of which are in phase I and have not entered phase 2, which may account for the lower transition rate. In addition, some drugs have problems of druggability, which were found in the early phase clinical trials, so the clinical trials cannot be progressed into the next phase. This result suggested that although the innovative drugs have been developed rapidly, there remains room for progress during the drug development in China.

## Conclusion

This study provides an overview of phase I clinical trial landscape during 2011–2020 in China. With the rising capability of clinical development and substantial policy support from the government, China has made rapid progress in drug development between 2011 and 2020. However, how to improve the innovation ability of the pharmaceutical enterprises and how to make good use of new study conceptions to improve the efficiency and success rate of phase I clinical trials are key issues that deserve consideration. More efforts need to be made for the Chinese pharmaceutical companies in order to leap from “imitation” to “innovation,” from “following” to “running side by side” to “leading.” Nonetheless, we believe that China has become a favorable location for innovative drug research and development and is readily prepared to contribute to global drug development.

## Data Availability Statement

The raw data supporting the conclusions of this article will be made available by the authors, without undue reservation.

## Author Contributions

XH worked with the conception/design. XZ, NL, SW, and HC helped in the methodology. Formal analysis was done by NL, SW, and CC. The manuscript was written by CC and NL. XH revised and edited the manuscript. All authors contributed to the article and approved the submitted version.

## Funding

This study was funded by the CAMS Innovation Fund for Medical Sciences (CIFMS 2021-I2M-1-003) and the China National Major Project for New Drug Innovation (2017ZX09304015 and 2019ZX09201-002).

## Conflict of Interest

The authors declare that the research was conducted in the absence of any commercial or financial relationships that could be construed as a potential conflict of interest.

## Publisher's Note

All claims expressed in this article are solely those of the authors and do not necessarily represent those of their affiliated organizations, or those of the publisher, the editors and the reviewers. Any product that may be evaluated in this article, or claim that may be made by its manufacturer, is not guaranteed or endorsed by the publisher.
